# Supplementary material for: The Construction and Exploration of a Comprehensive MicroRNA Centered Regulatory Network in Foxtail Millet (Setaria italica L.)
Source: Front Plant Sci. 2022 May 6;13:848474. doi: 10.3389/fpls.2022.848474 (PMC9121102; doi:10.3389/fpls.2022.848474)
Supplement: Supplementary file 2 [file Data_Sheet_2.zip › Sit-miR172a_Seita.5G265800.1_5938_TPlot.pdf]

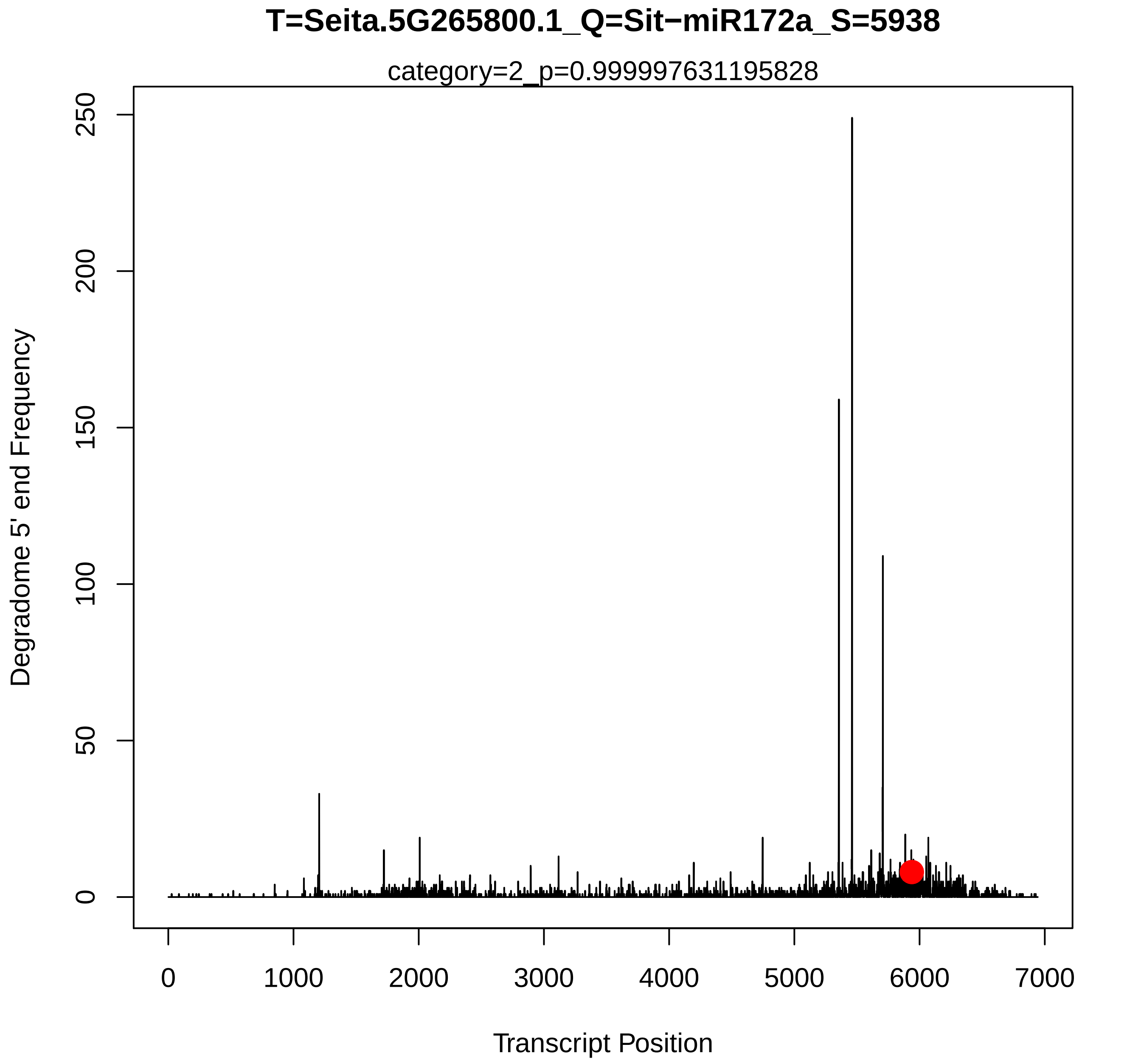

T=Seita.5G265800.1_Q=Sit−miR172a_S=5938
category=2_p=0.999997631195828
Degradome 5' end Frequency
0
50
100
150
200
250
0
1000
2000
3000
4000
5000
6000
7000
Transcript Position
